# CD133 prevents colon cancer cell death induced by serum deprivation through activation of Akt‐mediated protein synthesis and inhibition of apoptosis

**DOI:** 10.1002/2211-5463.13145

**Published:** 2021-03-28

**Authors:** Yusuke Mori, Ayaka Takeuchi, Kengo Miyagawa, Hiroyuki Yoda, Hiroaki Soda, Yoshihiro Nabeya, Naoko Watanabe, Toshinori Ozaki, Osamu Shimozato

**Affiliations:** ^1^ Laboratory of Oncogenomics Chiba Cancer Center Research Institute Japan; ^2^ Department of Biomolecular Science Faculty of Science Toho University Funabashi Japan; ^3^ Laboratory of Innovative Cancer Therapeutics Chiba Cancer Center Research Institute Japan; ^4^ Department of Esophago‐Gastrointestinal Surgery Chiba Cancer Center Hospital Japan

**Keywords:** Akt, CD133, colon carcinoma, resistance to nutritional stress

## Abstract

During the early phase of tumorigenesis, primary malignant cells survive within a low nutrition environment caused by a poorly organized vascular system. Here, we sought to determine the functional significance of CD133 in the survival of cancer cells under nutrient‐poor conditions. Knockdown and overexpression experiments demonstrated that CD133 suppresses colon cancer cell death induced by serum deprivation through activation of Akt‐mediated anti‐apoptosis and protein synthesis pathways. Furthermore, serum deprivation increased the amount of endogenous CD133 protein, which was regulated at least in part by phosphoinositide 3‐kinase. Thus, it is highly likely that CD133 contributes to the acquisition/maintenance of the resistance to stress arising from nutrient deficiency in early avascular tumor tissues.

## Graphical abstract

During tumorigenesis, primary malignant cells survive within a low nutrition environment as a result of a poorly organized vascular system. Here, we investigated the significance of CD133 in the survival of cancer cells under nutrient‐poor conditions. Our results suggest that CD133 contributes to the growth of tumors, in which nutrients are poorly supplied, through Akt‐mediated anti‐apoptosis and protein synthesis pathways.

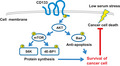

**Abbreviations**
4E‐BP1: eukaryotic translation initiation factor 4E‐binding protein 1
ANOVA: analysis of variance
CSC: cancer stem cell
DMEM: Dubecco’s modified Eagle’s medium
EV: empty vector
Ig: immunoglobulin
LY: LY294002
MAPK: mitogen‐activated protein kinase
mTOR: mechanistic target of rapamycin
OE: overexpression
PARP: poly(ADP‐ribose) polymerase
PI3K: phosphoinositide 3‐kinase
RT‐PCR: reverse transcription‐polymerase chain reaction
S6K: S6 kinase

Unlike normal tissues where the vascular system is well‐organized, tumor tissues are composed of massively proliferating malignant cells, which are exposed to the nutrient deficiency and the hypoxia as a result of their poorly organized vascular system such that tumor tissue necrosis occurs [1]. Treatment with the molecular‐targeted drugs, such as the neutralizing antibodies against the vascular endothelial growth factor and its receptor, is thus expected to have an anti‐tumor effect. These drugs might inhibit the supply of nutrients, as well as oxygen, via the attenuation of tumor angiogenesis [2]. Nevertheless, a theoretical root of cancer, so‐called cancer stem cells (CSCs), might survive and eventually develop into the primary malignant tumor tissues and recurrent/metastatic lesions. It is therefore suggested that CSCs are resistant to these stresses during the early stage of tumorigenesis. In good agreement with this notion, certain features of malignant tumor such as cellular migration and cell death inhibition were augmented in colorectal cancer cells, which express the stem cell‐related markers, under hypoxic and/or poor nutritional conditions [3, 4]. Exposure to hypoxia also conferred resistance to the anti‐tumor drugs and oxidative stress in brain tumor cells accompanied by an increase in stem cell‐related gene expression, such as *NOTCH*, *NANOG* and *BMI1* [5]. It is likely that these stem cell‐related gene products might enhance the abilities of cancerous cells to develop tumor tissues under the deleterious microenvironment. Therefore, it is expected that functional analysis of CSC markers will provide important insights into the attractive therapy targeting CSCs.

Recent reports have identified numerous cell surface antigens that express in the normal tissue stem cells, as well as CSCs, but not in the matured normal cells [6]. Among them, CD133, a type‐I membrane glycoprotein with a molecular weight of 120 kDa, was expressed in various normal tissues and thus has been considered to be the most reliable stem cell marker [7, 8]. It has also been suggested that CD133 acts as a promising candidate for CSC marker. For example, CD133‐positive tumor cells had higher abilities with respect to self‐renewal, tumorigenesis and resistance to treatment with anti‐tumor drugs and radiation compared to CD133‐negative tumor cells [9, 10, 11, 12]. The higher expression level of CD133 has been reported to be associated with the poor prognosis of the patients with lung, pancreas and colon cancers [13, 14, 15]. Although the extracellular activator(s) of CD133 are still unknown, its two tyrosine residues at 828 (Y828) and 858 (Y858) have been shown to be phosphorylated by proto‐oncogene products such as Src and FAK [16]. The phosphorylated‐CD133 (p‐CD133) activated the phosphoinositide 3‐kinase (PI3K)‐Akt signaling pathway in brain tumor cells, in which the p‐CD133 level was elevated dependent on their grade of malignancy [17]. Intriguingly, our previous studies revealed that a receptor tyrosine phosphatase PTPRK removes the phosphorus groups from these tyrosine residues of CD133, attenuates CD133‐mediated xenograft tumor growth of colorectal cancer cells in nude mice and stimulates anti‐tumor drug‐induced cell death [18, 19]. Given that CD133 participates in the induction of glucose uptake and autophagy in hepatocellular carcinoma cells in response to the lower glucose environment [20], it is possible that CD133 might potentiate the survival signals to adapt to the poor nutritional conditions within the progressing malignant tumor tissues. In the present study, we have established CD133‐knocked down and CD133‐overexpressing human colorectal cancer cells and then examined their proliferation and cell death rates under reduced concentrations of serum.

## Materials and methods

### Cell culture

Human colon cancer‐derived HT‐29, HCT116 and SW480 cells, as well as human embryonic kidney‐derived 293T cells, were cultured in Dulbecco's modified Eagle’s medium (DMEM) (Sigma‐Aldrich, St Louis, MO, USA) supplemented with 10% or 1% heat‐inactivated fetal bovine serum (Invitrogen, Carlsbad, CA, USA) and 50 µg·mL^−1^ penicillin/streptomycin (Sigma‐Aldrich) in a humidified atmosphere with 5% CO_2_ at 37 °C. Their identities were verified by a short tandem repeat assay.

### Forced expression and knockdown by lentiviral vectors

Lentivirus‐mediated transduction was performed as described previously [18, 19]. In brief, 293T cells were co‐transfected with combinations of the lentivirus packaging plasmids (MISSION Lentiviral packaging mix; Sigma‐Aldrich) together with the transducing plasmid carrying cDNA for wild‐type and amino acid‐substituted mutated CD133 (pCDH‐CMV‐MCS‐EF1‐Puro; System Biosciences, Mountain View, CA, USA), or for short hairpin RNA (shRNA) against *CD133* (pLKO.1; Sigma‐Aldrich) using FuGENE HD transfection reagent (Promega, Madison, WI, USA) in accordance with the manufacturer's instructions. Following the preparation of the cell‐free culture supernatants containing the virus vectors, the indicated colon cancer cells were cultured with the conditioned medium supplemented with 25% (v/v) of the virus‐containing culture supernatants for 24 h at 37 °C. These transfected cells were selected by puromycin (1 µg·mL^−1^; Sigma‐Aldrich).

### Semi‐quantitative RT‐PCR

Total RNA was extracted from the indicated cells using Isogen reagent (Nippon Gene, Tokyo, Japan) and 5 µg of total RNA was reverse‐transcribed by Superscript III reverse transcriptase (Invitrogen) in accordance with the manufacturer's instructions. The resultant cDNA was used for PCR. The oligonucleotide primer sets used were: *CD133*, 5ʹ‐TTCCAGAAGCTCTGAGGCAG‐3ʹ (forward) and 5ʹ‐AGAAATACCCCACCAGAGGC‐3ʹ (reverse); *GAPDH*, 5ʹ‐ACCACAGTCCATGCCATCAC‐3ʹ (forward) and 5ʹ‐TCCACCACCCTGTTGCTGTA‐3ʹ (reverse). *GAPDH* was used as an internal control. PCR products were separated on 1% agarose gels and visualized by ethidium bromide staining.

### Immunoblot analysis

Cells were lysed in a lysis buffer containing 50 mm Tris‐HCl (pH 7.5), 150 mm NaCl, 1% NP‐40, 1 mm EDTA and a protease inhibitor cocktail (Calbiochem, San Diego, CA, USA). Equal amounts of cell lysates were separated by SDS/PAGE under reduced conditions and electro‐transferred onto a poly(vinylidene difluoride) membrane (Merck Millipore, Billerica, MA, USA). The membrane was probed with primary antibodies against CD133 (W6C3B1; Miltenyi Biotec, Bergisch Gladbach, Germany), phospho‐Akt at Ser‐473 (#4060; Cell Signaling Technology, Beverly, MA, USA), Akt (#9272; Cell Signaling Technology), phospho‐Bad at Ser‐136 (#4366; Cell Signaling Technology), Bad (#9239; Cell Signaling Technology), caspase‐9 (#9502; Cell Signaling Technology), poly(ADP‐ribose) polymerase (PARP) (#9532; Cell Signaling Technology), mechanistic target of rapamycin (mTOR) (GT630198; Gene Tex, Inc., CA, USA), phospho‐mTOR at Ser‐2448 (GTX132803; GeneTex, Inc.) S6 kinase (S6K) (#2708; Cell Signaling Technology), phospho‐S6K at Thr‐389 (#9206; Cell Signaling Technology), eukaryotic translation initiation factor 4E‐binding protein 1 (4E‐BP1) (#9644; Cell Signaling Technology), phospho‐4E‐BP1 at Ser‐65 (#2855; Cell Signaling Technology) or with actin (A5060; Sigma‐Aldrich) followed by the incubation with the appropriate horseradish peroxidase‐conjugated anti‐mouse immunglobulin (Ig)G (#7074; Cell Signaling Technology) or with anti‐rabbit IgG antibody (#7076; Cell Signaling Technology). Immunoreactive signals were visualized using an ImmunoStar LD detection system (Wako, Tokyo, Japan) and then analyzed by ImageQuant LAS4000 mini Imager (GE Healthcare Bioscience, Pittsburgh, PA, USA) in accordance with the manufacturer’s instructions.

### Trypan blue dye exclusion assay

Cells were seeded into six‐well plates at a density of 5 × 10^4^ cells per well, and cultured for 5 days with DMEM medium supplemented with 10% or with 1% fetal bovine serum. Floating and adherent cells were collected and washed in ice‐cold 1× PBS. After brief centrifugation, cells were resuspended in fresh medium, mixed with equal volume of 0.4% trypan blue solution (Sigma‐Aldrich) and then analyzed using an automatic cell counter (TC20; Bio‐Rad, Hercules, CA, USA).

### Statistical analysis

The results are presented as the mean ± SD. Data were compared using an unpaired *t*‐test, one‐way analysis of variance (ANOVA) and a repeated‐measures two‐way ANOVA. Analyses were performed using ekuseru‐tokei 2010 (Social Survey Research Information Co., Ltd, Tokyo, Japan). *P* < 0.05 was considered statistically significant.

## Results

### Knockdown of *CD133* promotes colon cancer cell death in response to serum depletion

**Fig. 1:**
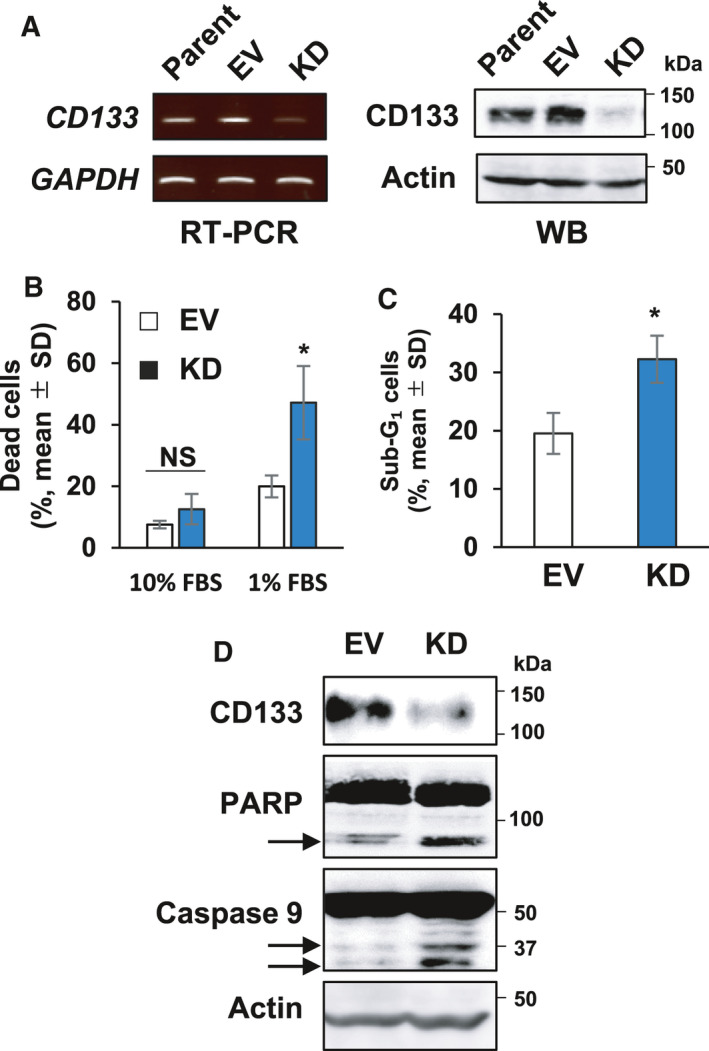
Knockdown of *CD133* augments the cell death of HCT116 cells under serum‐deprived conditions. (A) Establishment of *CD133*‐knocked down cells. Human colon cancer HCT116 (Parent) cells were infected with lentiviral vector harboring *CD133*‐targeting shRNA (HCT116/KD) or with empty control vector (HCT116/EV) and puromycin‐resistant cells were established. Total RNA and cell lysates prepared from the indicated cells were analyzed for CD133 expression by semi‐quantitative RT‐PCR (left) and immunoblotting (right), respectively. *GAPDH* or actin was used as an internal or loading control, respectively. (B) Trypan blue dye exclusion assay. HCT116/EV (EV, open bars) or HCT116/KD (KD, closed bars) cells were seeded into six‐well plates (50 000 cells per well) and cultured in the presence of 10% or 1% fetal bovine serum. Five days after cultivation, the floating and the attached cells were harvested and processed for a trypan blue assay. Data represent the mean ± SD (*n* = 3) and asterisks indicate a statistically significant difference (*P* < 0.05, *t*‐test). NS, not significant. (C) Flow cytometric analysis. HCT116/EV (EV) or HCT116/KD (KD) cells were cultured in the presence of 1% fetal bovine serum for 5 days. Floating and attached cells were collected and subjected to flow cytometry. The percentages of the attached cells with sub‐G1 DNA content are shown. Data represent the mean ± SD (*n* = 3). Asterisks indicate a statistically significant difference (*P* < 0.05, *t*‐test). (D) Immunoblot analysis. HCT116/EV (EV) or HCT116/KD (KD) cells were cultured in the presence of 1% fetal bovine serum for 5 days. Cell lysates (30 µg per lane) were prepared and analyzed by immunoblotting with the indicated antibodies. The arrows indicate cleaved PARP and caspase‐9. Actin was used as a loading control.

To examine the possible roles of CD133 in the cellular proliferation of colon cancer cells under nutrient‐deficient conditions, human colon cancer HCT116 cells were infected with a lentiviral vector carrying shRNA against *CD133* or with empty vector (EV). As shown in Fig. 1A, the endogenous CD133 level was successfully reduced by *CD133* shRNA at both the mRNA and protein levels. Because the morphologic features and proliferation rate of HCT116 cells were changed in the presence of 1% of serum compared to normal conditions (10% serum), we employed such conditions for subsequent studies (data not shown). We then cultivated *CD133*‐depleted (HCT116/KD), EV‐transduced (HCT116/EV) or parental HCT116 cells under serum‐deprived or normal conditions. At the indicated time points after starvation, the number of viable cells was examined via a WST assay. As shown in Fig. S1A,B, the number of viable HCT116/KD cells under serum‐deprived conditions was less than that of HCT116/EV and their parental cells, whereas HCT116/KD cells exhibited a proliferation rate similar to HCT116/EV and their parental cells under normal conditions.

These observations prompted us to investigate whether CD133 could be implicated in cell death induced by serum deprivation. Accordingly, HCT116 derivatives were exposed to serum deprivation for 5 days and the number of the dead cells was then scored using a trypan blue dye exclusion assay. As expected, the percentage of the trypan blue‐positive HCT116/KD cells was higher than that of HCT116/EV cells under serum‐deprived conditions (Fig. 1B). To further confirm these results, we carried out flow cytometric and immunoblot analyses. As shown in Fig. 1C, the number of HCT116/KD cells with sub‐G_1_ DNA content was higher than that of HCT116/EV cells exposed to serum starvation. Consistent with these observations, the proteolytic cleavage of both PARP and caspase‐9, which are reliable molecular markers of apoptotic cell death activation [21, 22], was strongly promoted in HCT116/KD cells following serum deprivation (Fig. 1D). Taken together, our results suggest that depletion of CD133 efficiently triggers the cell death of HCT116 cells in response to serum starvation.

### Forced expression of CD133 inhibits colon cancer cell death induced by serum deprivation

**Fig. 2:**
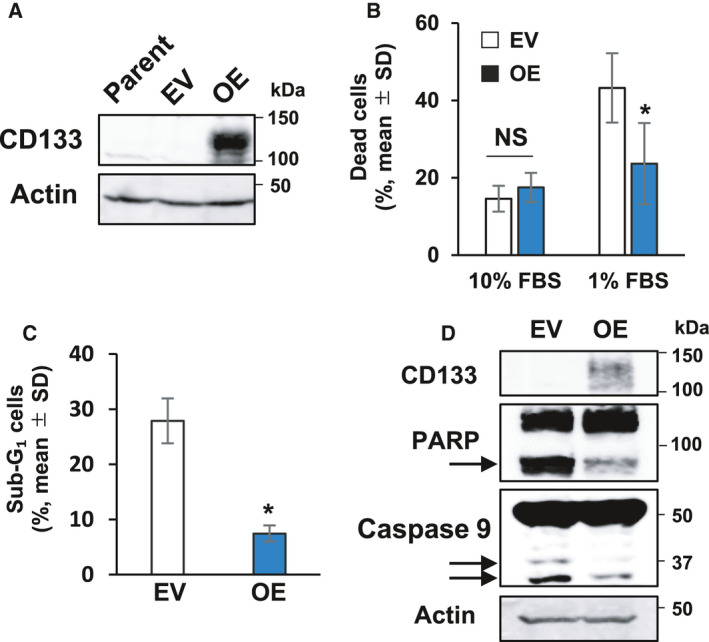
Forced expression of CD133 inhibits cell death of colon cancer cells in response to serum starvation. (A) Forced expression of CD133. CD133‐negative human colon cancer SW480 cells were transduced with lentiviral vector for wild‐type CD133 (SW480/OE) or with empty control vector (SW480/EV). Cell lysates were prepared from the parental SW480 cells and their derivatives and analyzed for CD133 expression by immunoblotting. Actin was used as a loading control. (B) Trypan blue dye exclusion assay. SW480/EV (open bars) or SW480/OE (closed bars) cells were cultured in the presence of 10% or 1% fetal bovine serum. Five days after treatment, floating and attached cells were harvested and processed for a trypan blue assay. Data represent the mean ± SD (*n* = 3) and the asterisk indicates a statistically significant difference (*P* < 0.05, *t*‐test). NS, not significant. (C) Flow cytometric analysis. SW480/EV or SW480/OE cells were cultured in the presence of 1% fetal bovine serum‐containing medium for 5 days. Floating and attached cells were collected and subjected to flow cytometry. The percentages of cells with sub‐G1 DNA content are shown. Data represent the mean ± SD (*n* = 3). An asterisk indicates a statistically significant difference (*P* < 0.05, *t*‐test). NS, not significant. (D) Immunoblot analysis. SW480/EV or SW480/OE cells were maintained in medium containing 1% fetal bovine serum for 3 days. Cell lysates (30 µg per lane) were prepared and analyzed by immunoblotting with the indicated antibodies. The arrows indicate cleaved PARP and caspase‐9. Actin was used as a loading control.

To further evaluate the possible roles of CD133 with respect to survival under nutrient‐deficient conditionds, we established other colon cancer SW480 cells stably overexpressing exogenous CD133 [SW480/overexpression (OE)] and EV‐transduced SW480 (SW480/EV) cells (Fig. 2A). SW480 derivatives were then cultured under normal or serum‐deprived conditions. At the indicated time points after serum starvation, the number of the viable cells was measured via a WST assay. As shown in Fig. S1C, SW480/OE cells continuously proliferated under serum‐deprived conditions, whereas the proliferation rates of SW480/EV and their parental cells slowed down in response to serum deprivation. Additionally, the proliferation rate of SW480/OE cells under normal conditions was largely identical to that of SW480/EV and their parental cells (Fig. S1D). To confirm the serum deprivation‐dependent cell death of SW480/OE cells, we performed a trypan blue dye exclusion assay. As shown in Fig. 2B, number of the trypan blue‐positive SW480/OE cells under serum‐deprived conditions was smaller than that of SW480/EV cells. In support of these results, the number of SW480/OE cells with sub‐G_1_ DNA content was smaller than that of SW480/EV cells (Fig. 2C). Consistent with these results, our immunoblot experiments clearly demonstrated that the proteolytic cleavage of both PARP and caspase‐9 is markedly prohibited in SW480/OE cells cultured under serum‐deprived conditions compared to SW480/EV cells (Fig. 2D). Collectively, these results indicate that CD133 has the ability to attenuate the serum starvation‐mediated cell death of colon cancer cells.

### CD133 potentiates the anti‐apoptotic activity of Bad through Akt activation in serum‐deprived colon cancer cells

**Fig. 3:**
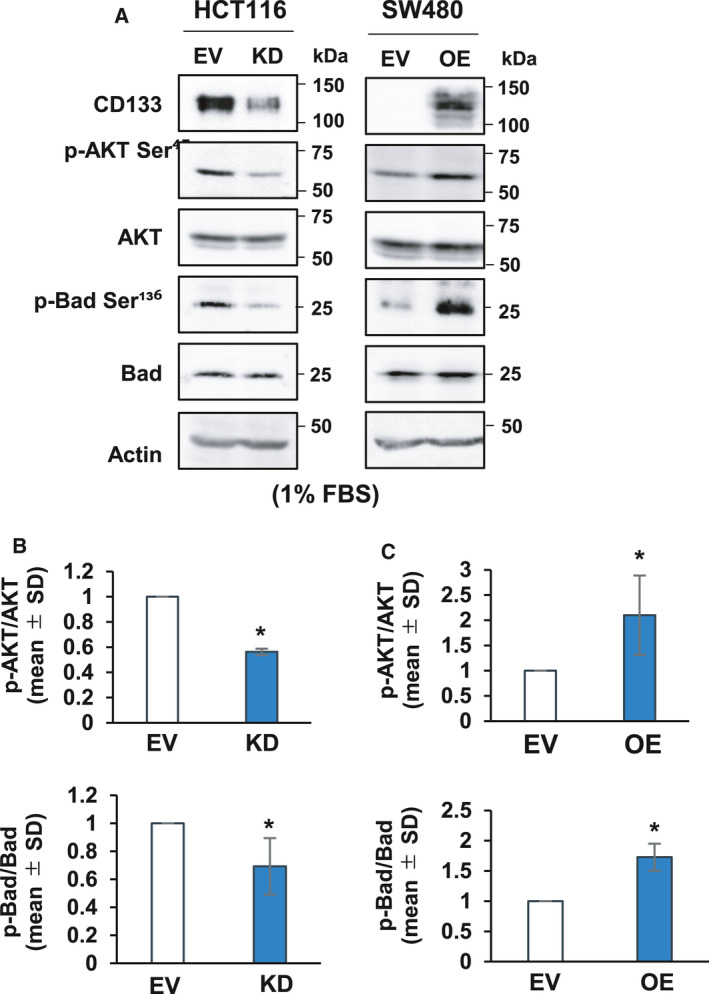
CD133 enhances the phosphorylation of Akt and its downstream target Bad following serum starvation. (A) Immunoblot analysis. The indicated cells were cultured in the presence of 1% fetal bovine serum for 5 days. Cell lysates (30 µg per lane) were prepared and processed for immunoblotting with the indicated antibodies. Actin was used as a loading control. (B, C) Relative band intensities of the phosphorylated proteins expressed in HCT116 derivatives (B) and SW480 derivatives (C) were determined based on three independent experiments and standardized with respect to total proteins. Data represent the mean ± SD (*n* = 3) and asterisks indicate a statistically significant difference (*P* < 0.05, *t*‐test).

Previously, we have reported that the CD133‐Akt axis plays a vital role in the promotion of colon cancer progression [18, 19]. Notably, it has been also reported that Akt inhibits cell death through the phosphorylation of an anti‐apoptotic protein Bad [23]. These findings prompted us to examine whether CD133 could stimulate the anti‐apoptotic Akt‐Bad pathway in serum‐starved colon cancer cells. For this purpose, HCT116/KD cells and HCT116/EV cells were exposed to the serum starvation for 5 days, and cell lysates were then analyzed for the phosphorylation status of Akt and Bad proteins by immunoblotting. As shown in Fig. 3A,B, the phosphorylation levels of Akt and Bad were significantly lower in HCT116/KD cells than in HCT116/EV cells. Based on these observations, we checked the phosphorylation levels of Akt and Bad in serum‐starved SW480/OE and SW480/EV cells. As shown in Fig. 3A,C, Akt and Bad were highly phosphorylated in SW480/OE cells compared to SW480/EV cells following serum deprivation. These results suggest that CD133 participates in the stimulation of the anti‐apoptotic Akt‐Bad pathway in response to serum deprivation.

### CD133 prevents serum deprivation‐induced colon cancer cell death through activation of translation effectors p70‐S6K and 4E‐BP1

**Fig. 4:**
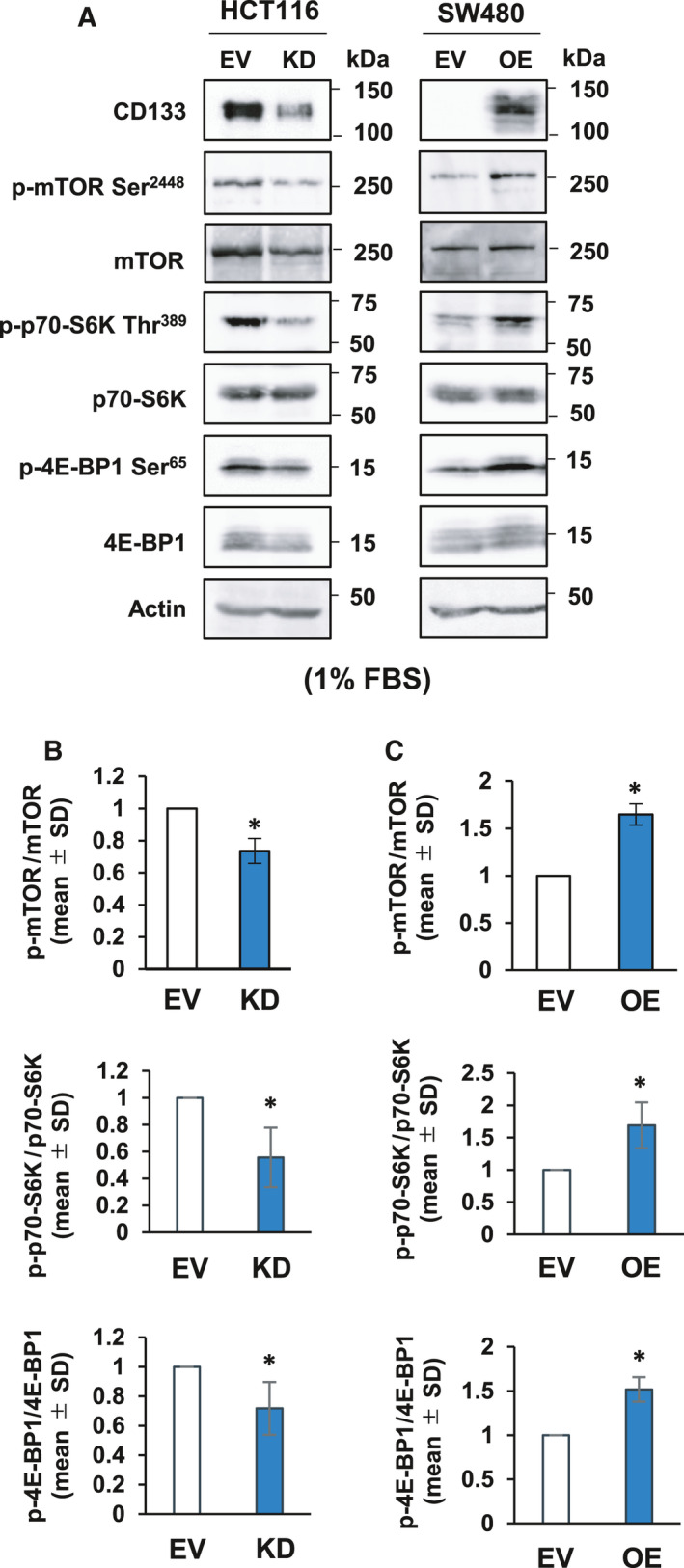
CD133‐mediated phosphorylation of protein synthesis‐related p70‐S6K and 4E‐BP1 in response to serum starvation. (A) Immunoblot analysis. The indicated colon cancer‐derived cells were cultured in the presence of 1% fetal bovine serum for 5 days. Cell lysates (30 µg per lane) were prepared and processed for immunoblotting with the indicated antibodies. Actin was used as a loading control. (B, C) Relative band intensities of the phosphorylated proteins standardized with respect to total proteins were determined based on three independent experiments. Data represent the mean ± SD (*n* = 3) and asterisks indicate a statistically significant difference (*P* < 0.05, *t*‐test).

Our results described above showed that SW480/OE cells continuously proliferate under serum‐deprived conditions (Fig. S1C). It has been reported that Akt, which is a downstream target of CD133, is implicated in survival and cell cycle progression under nutrient‐deficient conditions via the activation of mTOR, which regulates protein synthesis through phosphorylation of translational effectors, such as p70‐S6K and the eukaryotic initiation factor 4E (eIF4E)‐binding protein 1 (4E‐BP1) [24]. We therefore examined the phosphorylation levels of mTOR, p70‐S6K and 4E‐BP1 in HCT116/KD cells under serum‐deprived conditions. As shown in Fig. 4A,B, immunoblotting experiments revealed that the phosphorylation levels of mTOR at Ser‐2448, p70‐S6K at Thr‐389 and 4E‐BP1 at Ser‐65 in HCT116/KD cells are significantly reduced relative to those in HCT116/EV cells. To confirm these findings, their phosphorylation levels in serum‐starved SW480/OE and SW480/EV cells were also analyzed by immunoblotting. As shown in Fig. 4A,C, the phosphorylation levels of mTOR, p70‐S6K and 4E‐BP1 were remarkably elevated in SW480/OE cells compared to those in SW480/EV cells. These observations indicate that CD133 also tightly links to the Akt‐mTOR pathway, which might be implicated in the activation of protein synthesis through the phosphorylation of p70‐S6K and 4E‐BP1 following serum starvation.

### PI3K contributes to the serum deprivation‐mediated increase in CD133

**Fig. 5:**
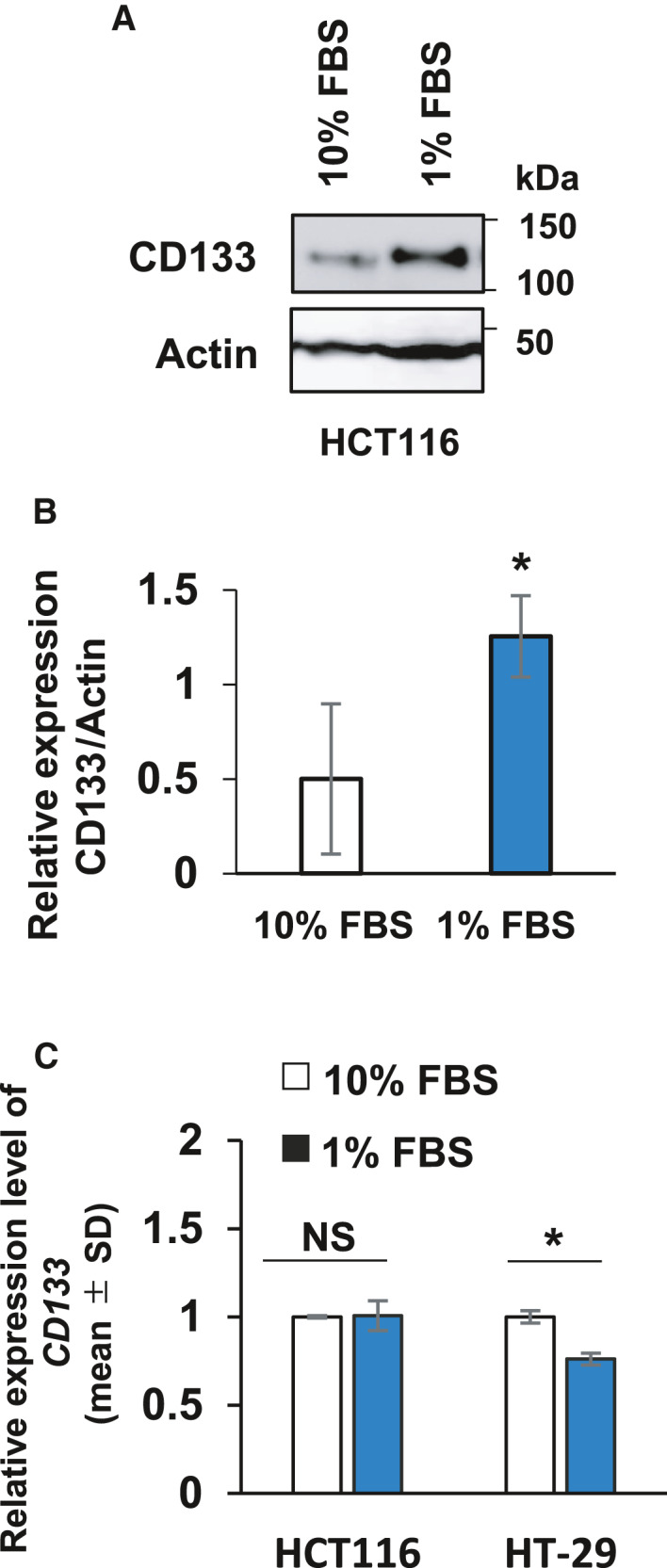
Up‐regulation of CD133 at the protein level following serum starvation. (A) Immunoblot analysis. HCT116 cells were cultured in medium containing 10% or 1% fetal bovine serum for 3 days. Cell lysates (30 µg per lane) were prepared and analyzed by immunoblotting with the indicated antibodies. Actin was used a loading control. (B) Relative band intensities of the phosphorylated proteins standardized with respect to total proteins were determined based on three independent experiments. Data represent the mean ± SD (*n* = 3) and asterisks indicate a statistically significant difference (*P* < 0.05, *t*‐test). (C) Real‐time RT‐PCR. Total RNA was extracted from the indicated cells cultured as in (A) and analyzed for *CD133* by real‐time RT‐PCR. The *CD133* level was normalized to the *GAPDH* level. Data represent the mean ± SD (*n* = 3) and asterisks indicate a statistically significant difference (*P* < 0.05, *t*‐test). NS, not significant.

Exposure to hyponutrition and hypoxia, which is often observed within the tumor microenvironment, caused an increase in endogenous CD133 in various tumor cells [4, 25, 26]. We therefore investigated the expression level of endogenous CD133 at the protein and mRNA levels in HCT116 cells cultured under serum‐deprived or normal conditions. As shown in Fig. 5A,B, the serum‐deprived HCT116 cells highly expressed CD133 protein compared to normal conditions. By contrast, the amount of *CD133* mRNA expressed in the serum‐deprived HCT116 cells was essentially comparable to that in cells under normal conditions (Fig. 5C), suggesting that CD133 expression is regulated at the post‐transcription level but not at the transcription level under serum‐deprived conditions.

**Fig. 6:**
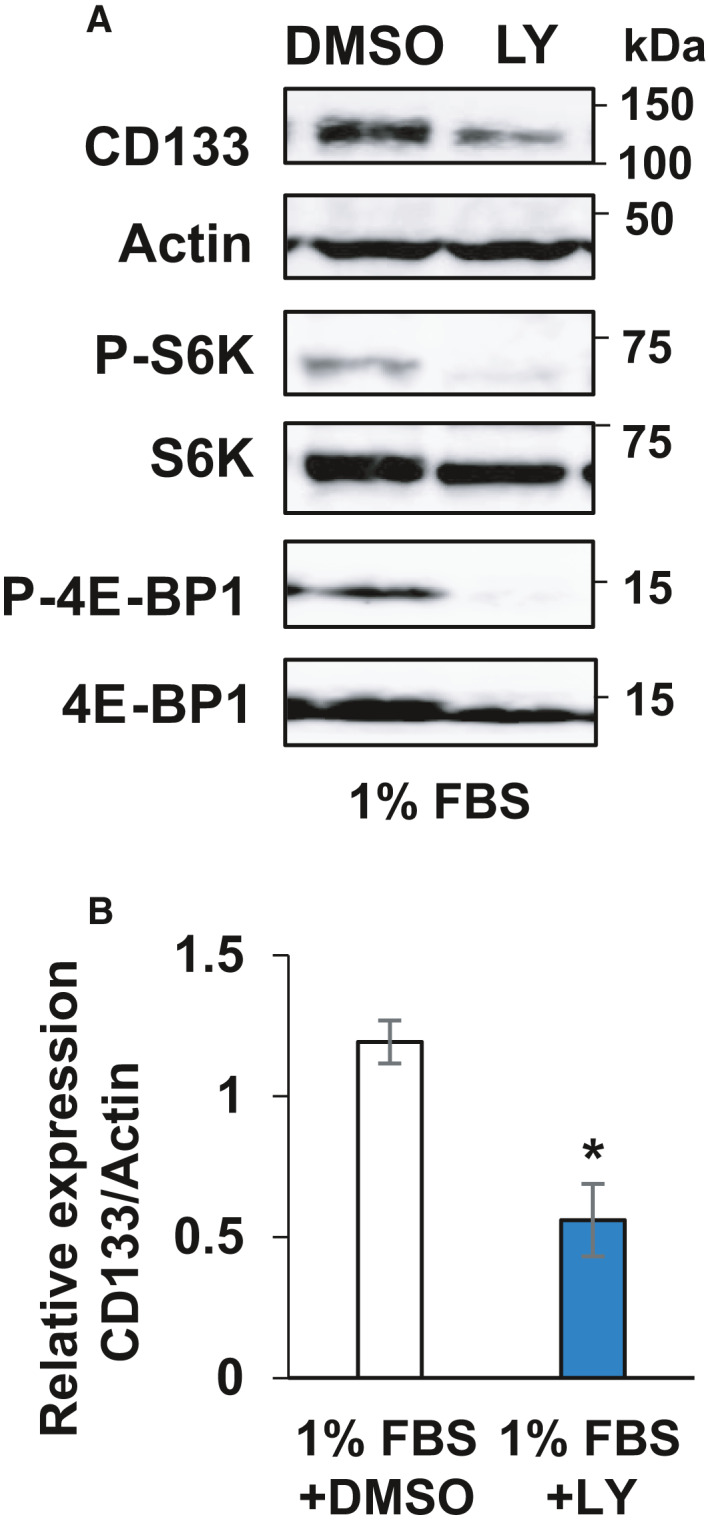
PI3K‐Akt pathway‐dependent regulation of CD133 in response to serum deprivation. (A) Immunoblot analysis. HCT116 cells were cultured in the presence of 1% fetal bovine serum for 3 days and then treated with PI3K inhibitor (LY294002) at 25 nm for 24 h. After treatment, cell lysates (30 µg per lane) were prepared and analyzed by immunoblotting with the indicated antibodies. Actin was used as a loading control. (B) Relative band intensities of the phosphorylated proteins standardized with respect to total proteins were determined based on three independent experiments. Data represent the mean ± SD (*n* = 3) and the asterisk indicates a statistically significant difference (*P* < 0.05, *t*‐test).

Because it has been shown that the PI3K/Akt pathway tightly links to CD133‐mediated survival signaling and also regulates protein synthesis [17], we aimed to address the possible roles of the PI3K/Akt pathway in the regulation of CD133, S6K and 4E‐BP1. To this end, HCT116 cells were treated with a PI3K inhibitor, LY294002 (LY), under lower serum conditions. Cell lysates were prepared and analyzed for CD133 by immunoblotting. As shown in Fig. 6A,B, LY treatment attenuated the phosphorylation of both S6K and 4E‐BP1, which was accompanied by an obvious reduction of CD133.

## Discussion

A growing body of evidence suggests that a putative root of cancer, CSC, survives during the early phase of tumorigenesis and/or metastasis. The extensively proliferating tumor cells survive under deleterious conditions with insufficient amounts of nutrients and oxygen, which is a result of the poorly organized vascular system [27]. With respect to tumorigenesis, Zhu *et al*. [28] reported that CD133‐expressing tumor cells acquire a higher oncogenic ability in response to genetic insults. However, the precise molecular mechanisms underlying the resistance of CSC to these stresses have not been fully clarified. In the present study, we examined how CD133 could contribute to the acquisition of resistance to undernutrition in colon cancer cells. Our present findings have demonstrated that, in support of previous investigations, CD133 promotes the survival of colon cancer cells under serum‐deprived conditions through activation of Akt [18, 19]. It is worth noting that the CD133‐Akt axis stimulates not only the anti‐apoptotic Bad protein, but also the initiation of protein synthesis. As described previously, p70‐S6K phosphorylated Bad at Ser‐136 and thus suppressed apoptotic cell death [29]. Taken together, the findings of the present study strongly suggest that the CD133‐Akt axis plays a crucial role in the prevention of serum starvation‐induced colon cancer cell death and is responsible for colon cancer development. Additionally, we observed up‐regulation of CSC‐related genes such as *OCT4* and *NANOG* in *CD133*‐positive HCT116 cells but not in *CD133*‐depleted HCT116 cells when exposed to undernutrition (Fig. S2). The results of the present study support the possibility that CD133 potentiates typical CSC‐like characteristics, such as sphere formation [18] and drug resistance [19]. Despite massive CD133 production in the cytosol, the amount of membrane‐bound CD133 in serum‐deprived HCT116 cells was similar to that in cells left untreated (Fig. S3A,B). In good agreement with our observations, previous clinicopathological studies have demonstrated that CD133 protein is often detected in both the membrane and cytosol of cancer cells in various tumor tissues [30, 31]. Although the ligand of CD133 is still unknown, it is conceivable that cytosolic CD133 also plays a role in the regulation of the acquisition and/or maintenance of CSC‐characteristics. With this in mind, we aimed to further determine how the membrane‐bound CD133 and/or the cytosolic CD133 could regulate CSC characteristics at least in part through Akt activation.

Meanwhile, we previously found that CD133 enhances the phosphorylation of p38 mitogen‐activated protein kinase (MAPK) in neuroblastoma cells [32]. p38 MAPK was shown to be involved in the apoptotic pathway in response to the various stress stimuli [33]. Previous studies have demonstrated that there is cross‐talk between the p38 MAPK and Akt pathways during myoblast differentiation [34] and epithelial and mesenchymal transition in non‐small cell lung carcinoma [35]. However, the phosphorylation levels of p38 MAPK remained unchanged in serum‐deprived colon cancer HCT116 and SW480 cells, regardless of their CD133 expression levels (Fig. S4). Similar results were also obtained in hepatocellular carcinoma cells [36]. Consistent with our previous observations [18, 19], Akt activity tightly is linked to the intracellular carboxyl terminal tyrosine phosphorylation of CD133 even in serum‐starved cells (Fig. S5). Thus, it is likely that activation of the CD133‐p38 MAPK pathway depends on the cellular context, although this remains to be determined.

Another important finding of the present study is that the PI3K‐Akt pathway participates in the regulation of CD133 at the protein level. According to the previous studies, CD133 was increased in cells exposed to numerous stresses, such as hypoxia and serum starvation [3, 25]; however, the molecular mechanisms behind these phenomena have remained elusive. In the present study, we found for the first time that undernutrition causes an increase in CD133 at the protein level but not at the transcriptional level, which is efficiently blocked by PI3K inhibitor LY294002. Because Akt and its downstream target mTOR were activated in serum‐starved CD133‐expressing colon cancer cells, it is possible that the CD133‐Akt‐mTOR regulatory axis positively regulates CD133 production through augmentation of protein synthesis‐related p70‐S6K and 4E‐BP1 [37]. Of note, the half‐life of CD133 was unaffected by serum starvation (Fig. S6). In accordance with our observations, Soeda *et al*.[38] reported that the activation of PI3K and/or mTOR up‐regulates CD133 in brain tumor cells under hypoxic conditions. By contrast, Matsumoto *et al*.[39] demonstrated that inhibition of PI3K and mTOR under hypoxic conditions up‐regulates the CD133 protein level in other colon cancer‐derived WiDr cells. Despite observations showing that hypoxic conditions up‐regulate CD133 at the protein level but not at the mRNA level in HCT116 cells (Fig. S7), the molecular mechanisms regulating CD133 protein level in response to hypoxia still remain controversial. However, it has been shown that glucose deficiency stimulates *CD133* transcription in other colon cancer SW620 cells [25]. Unfortunately, we could not examine the effects of glucose starvation on CD133 mRNA expression under our experimental system because our HCT116 and SW480 cells lack tolerability to glucose‐deprivation (data not shown). Further studies are required to adequately assess how the stresses arising from the tumor microenvironment could up‐regulate CD133 at the protein level.

**Fig. 7:**
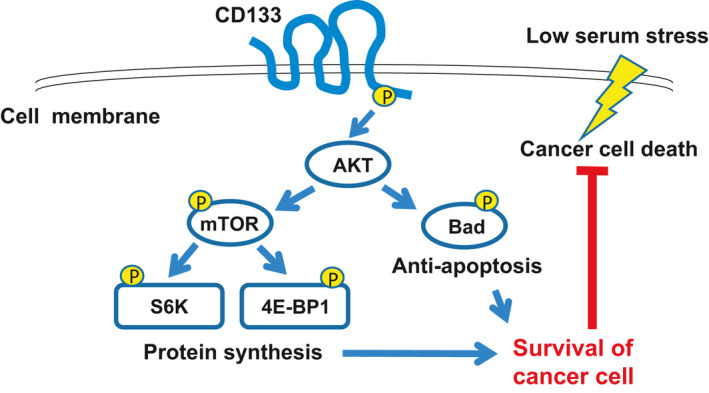
Summary of the present study.

In conclusion, our current observations strongly suggest that, upon under nutritional stress, CD133 potentiates the survival signals originating from Akt‐mediated activation of both the anti‐apoptosis and pro‐protein synthesis pathways (Fig. 7). Our present findings also provide a clue to understanding the functional significance of CD133 during the early phase of tumorigenesis and metastasis.

## Conflict of interest

The authors declare that they have no conflict of interest.

### Author contributions

OS conceived and supervised the study. YM and OS designed the experiments. YM, AT, KM and HY performed the experiments. OS, NW, HS, YN and TO analyzed and interpreted data. YM, TO and OS wrote the manuscript.

## Supporting information


**Fig. S1.** *In vitro* proliferation of CD133‐depleted or CD133‐overexpressing colon cancer cells under normal conditions. The indicated HCT116 derivatives (A and B) or SW480 derivatives (C and D) were seeded into 96‐well plates at a density of 500 cells per well, and allowed to attach the bottoms of culture plates overnight. The cells were further cultured for 5 days in the presence of 1% (A and C) or 10% fetal bovine serum (B and D). At the indicated time points, cell viability was examined using Cell Counting Kit‐8 reagent (Dojindo Molecular Technologies, Rockville, MD, USA) in accordance with the manufacturer’s instructions. The results represent the mean ± SD (*n* = 4) and asterisks indicate a statistically significant difference compared to mock‐transduced (EV) cells (*P* < 0.05, repeated‐measures two‐way ANOVA). NS, not significant.

**Fig. S2.** Expression level of stem cell‐related genes in serum‐starved HCT116 cells. HCT116/EV and HCT116/KD cells were cultured for 24 h in medium containing 10% or 1% fetal bovine serum. Total RNA was extracted from cells using Isogen reagent (Nippon Gene). The first‐strand cDNA was synthesized from 1 µg of total RNA using ReverTra Ace reagent (Toyobo, Osaka, Japan) and then subjected to quantitative RT‐PCR with Thunderbird reagent in accordance with the manufacturer’s instructions (Toyobo). Relative expression levels of target genes were evaluated by the 2^‐ΔΔ^ *^CT^* method compared to the level of *GAPDH* in HCT116/EV cells cultured with 10% fetal bovine serum‐containing medium as a reference sample. Data are the mean ± SD of three independent experiments and asterisks indicate a statistically significant difference compared to HCT116/EV cells (*P < 0.05, one‐way ANOVA). NS, not significant. The oligonucleotide primer sets used were: *OCT4*, 5ʹ‐GTACTCCTCGGTCCCTTTCC‐3ʹ (forward) and 5ʹ‐CAAAAACCCTGGCACAAACT‐3ʹ (reverse); NANOG, 5ʹ‐TTCCTTCCTCCATGGATCTG‐3ʹ (forward) and 5ʹ‐TCTGCTGGAGGCTGAGGTAT‐3ʹ (Reverse); *GAPDH*, 5ʹ‐ATGGAAATCCCATCACCATCTT‐3ʹ (forward) and 5ʹ‐CGCCCCACTTGATTTTGG‐3ʹ (reverse).

**Fig. S3.** Expression level of membrane‐bound CD133 in serum‐deprived HCT116 cells. (A) Flow cytometry. HCT116 cells were cultured for 3 days in medium containing 10% or 1% fetal bovine serum. The cells were stained for 30 min with phycoerythrin‐conjugated mouse anti‐CD133 monoclonal antibody (293C3; Miltenyi Biotec) or the corresponding isotype control mouse IgG (eBGM2b; eBioscience, San Diego, CA, USA) at 4 °C. The CD133 level on the cell surface was analyzed using a FACSCalibur flow cytometer (BD Biosciences, Franklin Lakes, NJ, USA) and flowjo software (Tree Star, Ashland, OR, USA). A representative histogram is shown. (B) Immunofluorescent analysis. HCT116 cells were cultured on the coverslips for 3 days with DMEM medium supplemented with 10% or 1% fetal bovine serum. Cells were fixed with 3.7% formaldehyde/PBS and permeabilized by 0.1% Triton X‐100/PBS. Cells were then stained with rabbit anti‐CD133 monoclonal antibody (D2V8Q; Cell Signaling Technology, dilution 1 : 400) followed by Alexa 488‐conjugated anti‐rabbit IgG (Sigma‐Aldrich). The coverslips were mounted on the slide glasses with ProLong™ Gold Antifade Mountant with DAPI (Thermo Fisher Scientific, Waltham, MA, USA), and fluorescein images were captured via confocal laser microscopy. Representative images of DAPI, Alexa 488 and the merged image are shown. Scale bars = 50 µm. Mean fluorescence intensity of CD133 is summarized on the right. *P < 0.05 (*t*‐test, *n* = 4).

**Fig. S4.** The activated Akt has an undetectable effect on the phosphorylation level of p38 MAPK. Immunoblot analysis. The indicated cells were cultured in 1% fetal bovine serum‐containing medium for 3 days. Cell lysates (50 µg per lane) were prepared and processed for immunoblotting with the indicated antibodies. Actin was used as a loading control.

**Fig. S5.** Tyrosine phosphorylation of CD133 suppresses cell death in response to serum deprivation through activation of the Akt‐Bad pathway. (A) Forced expression of CD133 and its amino acid‐substituted mutants. SW480 cells were transduced with the lentiviral vector for wild‐type CD133 (SW480/OE), a phenylalanine‐substituted CD133 mutant (SW480/FF), a glutamate‐substituted CD133 mutant (SW480/EE) or with empty control vector (SW480/EV) and, finally, puromycin‐resistant cells were established. Their expression levels were checked by flow cytometry. Representative histograms are shown. (B) Trypan blue dye exclusion assay. SW480/EV (EV, open bars) SW480/OE (OE, grey bars) SW480/FF (FF, hatched bars) or SW480/EE (EE, closed bars) cells were cultured in medium containing 10% or 1% fetal bovine serum. Five days after cultivation, floating and attached cells were harvested and processed for a trypan blue assay. Data represent the mean ± SD (*n* = 3) and asterisks indicate a statistically significant difference (*P* < 0.05, ANOVA). NS, not significant. (C and D) Immunoblot analysis. The indicated cells were cultured in the presence of 1% fetal bovine serum for 3 days. Cell lysates (30 µg per lane) were prepared from floating plus attached cells (C) or from attached cells (D) and then processed for immunoblotting with the indicated antibodies. Arrows indicate cleaved PARP and caspase‐9 (C). Actin was used as a loading control.

**Fig. S6.** Serum deprivation has an undetectable effect on the half‐life of CD133. Cycloheximide block. HCT116 cells cultured in 10% fetal bovine serum‐ or 1% fetal bovine serum‐containing medium were treated with 100 µg·mL^−1^ cycloheximide (CHX). At the indicated time points after treatment, cell lysates (35 µg per lane) were prepared and subjected to immunoblotting with the indicated antibodies. Actin was used as a loading control.

**Fig. S7.** Hypoxia increases the CD133 level in HCT116 cells at the protein level. (A) CD133 level under hypoxia. HCT116 (6 × 10^5^ cells per dish) cells were seeded in 6‐cm culture dishes with 10% fetal bovine serum/DMEM and then cultured under hypoxic conditions (0.1% O_2_ and 5% CO_2_) using an AnaeroPack (Mitsubishi Gas Chemical, Tokyo, Japan). Twenty‐four hours after incubation, cells were lysed with a lysis buffer containing 50 mm Tris‐HCl (pH 7.5), 150 mm NaCl, 1% NP‐40, 1 mm EDTA and a protease inhibitor cocktail (Calbiochem) and the CD133 level was analyzed by immunoblot analysis with antibodies against CD133 (W6C3B1; Miltenyi Biotec) followed by horseradish peroxidase‐conjugated anti‐mouse IgG (#7074; Cell Signaling Technology) and visualized as described in the Materials and methods. (B) *CD133* mRNA level under hypoxic conditions. HCT116 cells were exposed to hypoxic conditions for 24 h as described in (A). Total RNA was extracted from the cells using Isogen reagent (Nippon Gene). The first‐strand cDNA was synthesized from 1 µg of total RNA using ReverTra Ace reagent (Toyobo, Osaka, Japan) and then subjected to quantitative RT‐PCR with Thunderbird reagent in accordance with the manufacturer’s instructions (Toyobo). Relative expression levels of target genes were evaluated by the 2^‐ΔΔCT^ method compared to the level of *GAPDH* in HCT116 cells under normoxia as a reference sample. Data show the mean ± SD of three independent experiments and asterisks indicate a statistically significant difference compared to HCT116/EV cells (*P < 0.05, *t*‐test). NS, not significant. The oligonucleotide primer sets used were: *CD133*, 5ʹ‐ATCTGCAGTGGATCGAGTTCTCT‐3ʹ (forward) and 5ʹ‐GCGGTGGCCACAGGTTT‐3ʹ (reverse); GLUT, 5ʹ‐CTTCACTGTCGTGTCGCTGT‐3ʹ (forward) and 5ʹ‐CCAGGACCCACTTCAAAGAA‐3ʹ (Reverse); *GAPDH*, 5ʹ‐ATGGAAATCCCATCACCATCTT‐3ʹ (forward) and 5ʹ‐CGCCCCACTTGATTTTGG‐3ʹ (reverse).

## Data Availability

The data that support the findings of this study are available in the [Link], [Link], [Link], [Link], [Link], [Link], [Link] of this article.
