# Supplementary material for: CD133 prevents colon cancer cell death induced by serum deprivation through activation of Akt‐mediated protein synthesis and inhibition of apoptosis
Source: FEBS Open Bio. 2021 Mar 28;11(5):1382–94. doi: 10.1002/2211-5463.13145 (PMC8091590; doi:10.1002/2211-5463.13145)
Supplement: Supplementary file 1 — Fig. S1. In vitro proliferation of CD133‐depleted or CD133‐overexpressing colon cancer cells under normal conditions. The indicated HCT116 derivatives (A and B) or SW480 derivatives (C and D) were seeded into 96‐well plates at a density of 500 cells per well, and allowed to attach the bottoms of culture plates overnight. The cells were further cultured for 5 days in the presence of 1% (A and C) or 10% fetal bovine serum (B and D). At the indicated time points, cell viability was examined using Cell Counting Kit‐8 reagent (Dojindo Molecular Technologies, Rockville, MD, USA) in accordance with the manufacturer’s instructions. The results represent the mean ± SD (n = 4) and asterisks indicate a statistically significant difference compared to mock‐transduced (EV) cells (P < 0.05, repeated‐measures two‐way ANOVA). NS, not significant. [file FEB4-11-1382-s003.pptx]

## Slide 1
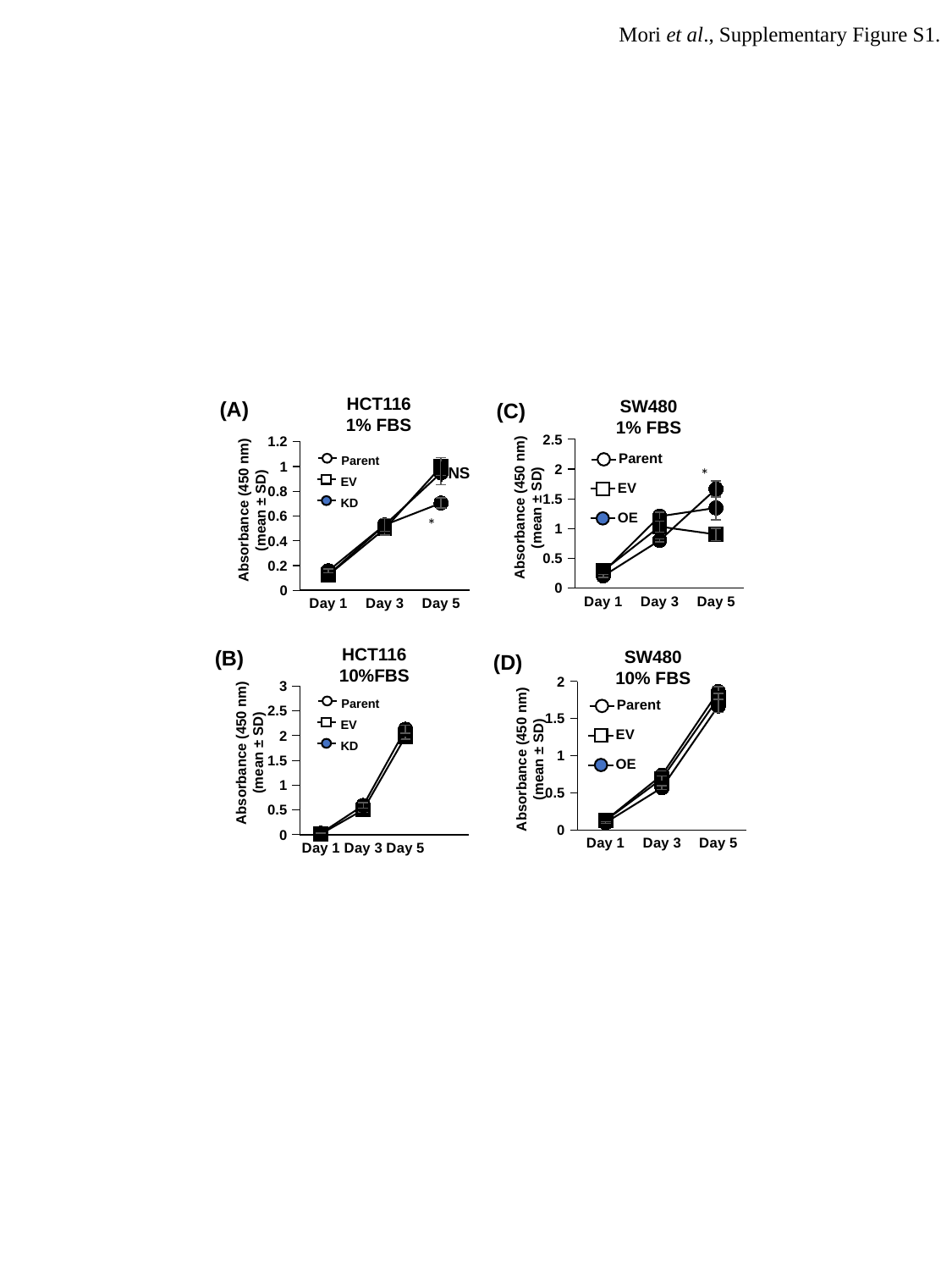

Mori et al., Supplementary Figure S1.
HCT116
1% FBS
SW480
1% FBS
(A)
(C)
### Chart
| Category | parent | pCDH | CD133-OE |
|---|---|---|---|
| Day 1 | 0.267 | 0.2895 | 0.20199999999999999 |
| Day 3 | 1.2060000000000002 | 1.0317500000000002 | 0.79975 |
| Day 5 | 1.347 | 0.8997499999999999 | 1.666 |
### Chart
| Category | parent | pLKO.1 | CD133-KD |
|---|---|---|---|
| Day 1 | 0.1255 | 0.12275 | 0.15775 |
| Day 3 | 0.52675 | 0.49625 | 0.52775 |
| Day 5 | 0.9487500000000001 | 0.9984999999999999 | 0.705 |Parent
EV
OE
Parent
EV
KD
NS
*
Absorbance (450 nm)
(mean ± SD)
Absorbance (450 nm)
(mean ± SD)
*
HCT116
10%FBS
(B)
SW480
10% FBS
(D)
### Chart
| Category | parent | pCDH | CD133-OE |
|---|---|---|---|
| Day 1 | 0.13025 | 0.12875 | 0.09874999999999998 |
| Day 3 | 0.73875 | 0.688 | 0.56875 |
| Day 5 | 1.8619999999999999 | 1.775 | 1.6687499999999997 |
### Chart
| Category | Parent | pLKO.1 | sh144 |
|---|---|---|---|
| Day 1 | 0.014500000000000013 | 0.016250000000000014 | 0.02999999999999997 |
| Day 3 | 0.565 | 0.50325 | 0.5912499999999999 |
| Day 5 | 1.7905000000000002 | 1.9817500000000001 | 2.1295 |Parent
EV
KD
Parent
EV
Absorbance (450 nm)
(mean ± SD)
Absorbance (450 nm)
(mean ± SD)
OE
