# Supplementary material for: CD133 prevents colon cancer cell death induced by serum deprivation through activation of Akt‐mediated protein synthesis and inhibition of apoptosis
Source: FEBS Open Bio. 2021 Mar 28;11(5):1382–94. doi: 10.1002/2211-5463.13145 (PMC8091590; doi:10.1002/2211-5463.13145)
Supplement: Supplementary file 2 — Fig. S2. Expression level of stem cell‐related genes in serum‐starved HCT116 cells. HCT116/EV and HCT116/KD cells were cultured for 24 h in medium containing 10% or 1% fetal bovine serum. Total RNA was extracted from cells using Isogen reagent (Nippon Gene). The first‐strand cDNA was synthesized from 1 µg of total RNA using ReverTra Ace reagent (Toyobo, Osaka, Japan) and then subjected to quantitative RT‐PCR with Thunderbird reagent in accordance with the manufacturer’s instructions (Toyobo). Relative expression levels of target genes were evaluated by the 2‐ΔΔ CT method compared to the level of GAPDH in HCT116/EV cells cultured with 10% fetal bovine serum‐containing medium as a reference sample. Data are the mean ± SD of three independent experiments and asterisks indicate a statistically significant difference compared to HCT116/EV cells (*P < 0.05, one‐way ANOVA). NS, not significant. The oligonucleotide primer sets used were: OCT4, 5ʹ‐GTACTCCTCGGTCCCTTTCC‐3ʹ (forward) and 5ʹ‐CAAAAACCCTGGCACAAACT‐3ʹ (reverse); NANOG, 5ʹ‐TTCCTTCCTCCATGGATCTG‐3ʹ (forward) and 5ʹ‐TCTGCTGGAGGCTGAGGTAT‐3ʹ (Reverse); GAPDH, 5ʹ‐ATGGAAATCCCATCACCATCTT‐3ʹ (forward) and 5ʹ‐CGCCCCACTTGATTTTGG‐3ʹ (reverse). [file FEB4-11-1382-s007.pptx]

## Slide 1
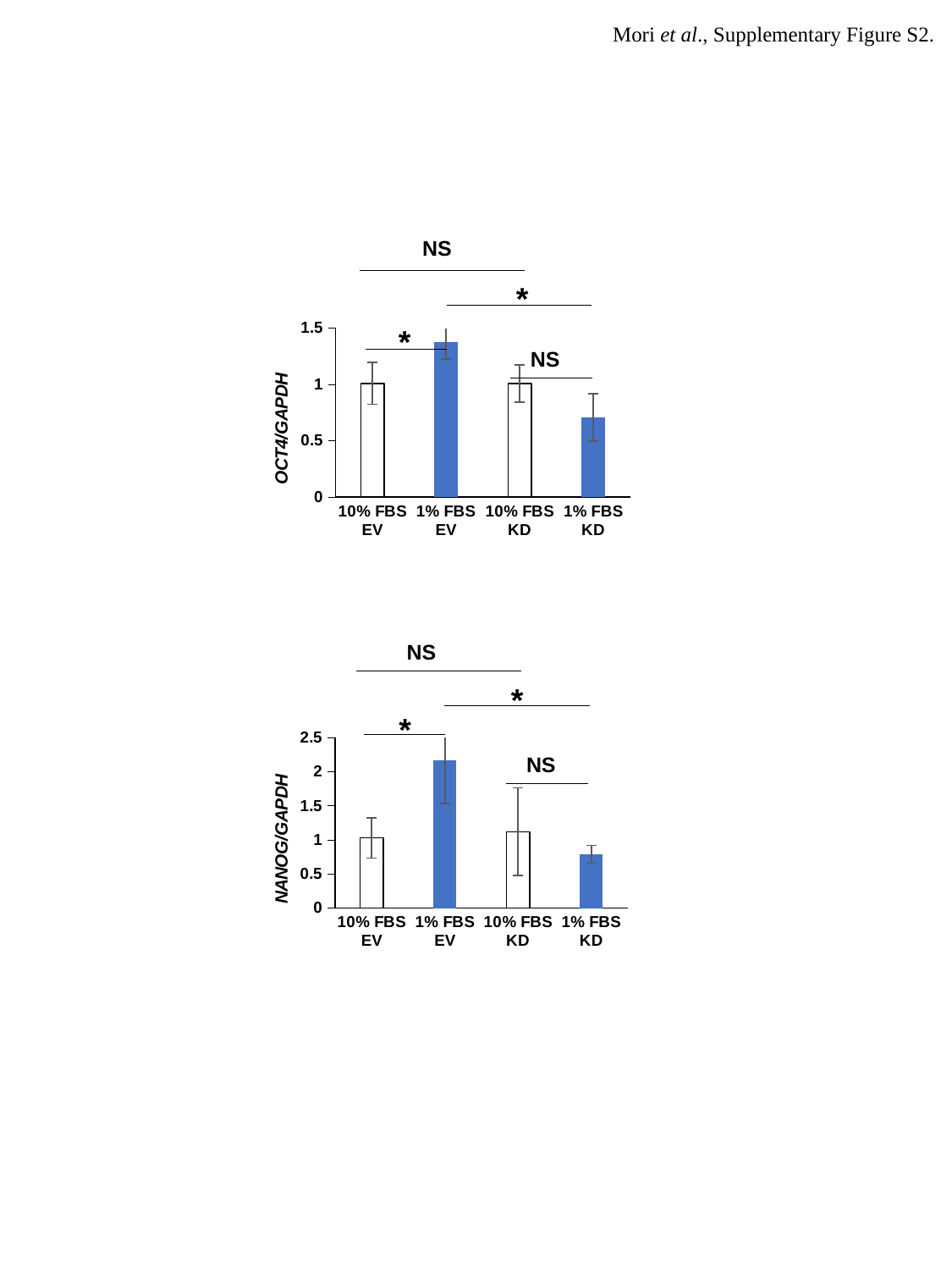

Mori et al., Supplementary Figure S2.
NS
*
### Chart
| Category | |
|---|---|
| 10% FBS
EV | 1.0107967529849864 |
| 1% FBS
EV | 1.3756045416885023 |
| 10% FBS
KD | 1.0095781342623293 |
| 1% FBS
KD | 0.7089281816881328 |*
NS
NS
*
*
### Chart
| Category | |
|---|---|
| 10% FBS
EV | 1.0307727182064221 |
| 1% FBS
EV | 2.16853924989759 |
| 10% FBS
KD | 1.1201306569489808 |
| 1% FBS
KD | 0.7914059248407196 |NS
