# Supplementary material for: CD133 prevents colon cancer cell death induced by serum deprivation through activation of Akt‐mediated protein synthesis and inhibition of apoptosis
Source: FEBS Open Bio. 2021 Mar 28;11(5):1382–94. doi: 10.1002/2211-5463.13145 (PMC8091590; doi:10.1002/2211-5463.13145)
Supplement: Supplementary file 3 — Fig. S3. Expression level of membrane‐bound CD133 in serum‐deprived HCT116 cells. (A) Flow cytometry. HCT116 cells were cultured for 3 days in medium containing 10% or 1% fetal bovine serum. The cells were stained for 30 min with phycoerythrin‐conjugated mouse anti‐CD133 monoclonal antibody (293C3; Miltenyi Biotec) or the corresponding isotype control mouse IgG (eBGM2b; eBioscience, San Diego, CA, USA) at 4 °C. The CD133 level on the cell surface was analyzed using a FACSCalibur flow cytometer (BD Biosciences, Franklin Lakes, NJ, USA) and flowjo software (Tree Star, Ashland, OR, USA). A representative histogram is shown. (B) Immunofluorescent analysis. HCT116 cells were cultured on the coverslips for 3 days with DMEM medium supplemented with 10% or 1% fetal bovine serum. Cells were fixed with 3.7% formaldehyde/PBS and permeabilized by 0.1% Triton X‐100/PBS. Cells were then stained with rabbit anti‐CD133 monoclonal antibody (D2V8Q; Cell Signaling Technology, dilution 1 : 400) followed by Alexa 488‐conjugated anti‐rabbit IgG (Sigma‐Aldrich). The coverslips were mounted on the slide glasses with ProLong™ Gold Antifade Mountant with DAPI (Thermo Fisher Scientific, Waltham, MA, USA), and fluorescein images were captured via confocal laser microscopy. Representative images of DAPI, Alexa 488 and the merged image are shown. Scale bars = 50 µm. Mean fluorescence intensity of CD133 is summarized on the right. *P < 0.05 (t‐test, n = 4). [file FEB4-11-1382-s006.pptx]

## Slide 1
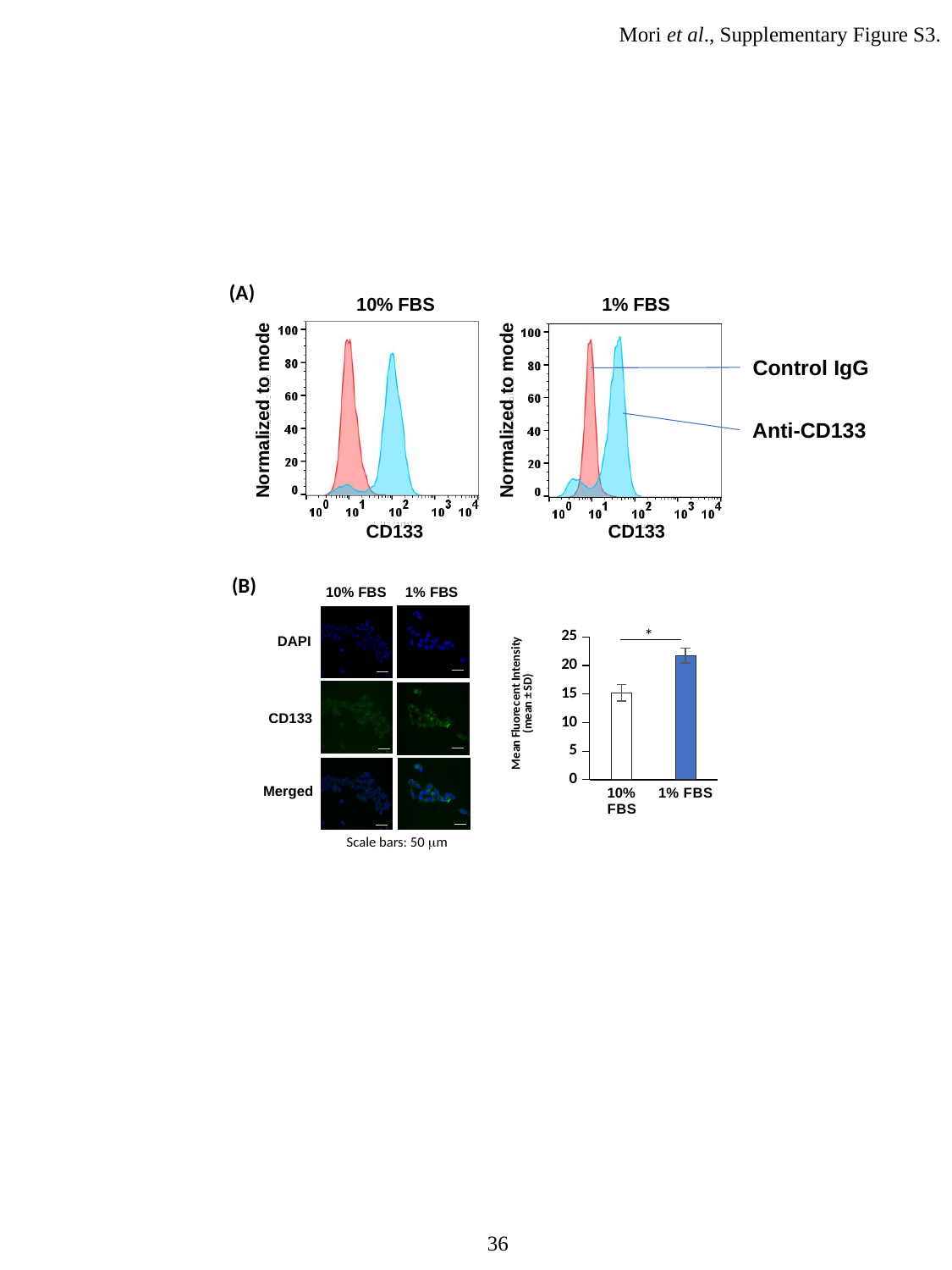

Mori et al., Supplementary Figure S3.
(A)
10% FBS
1% FBS
Control IgG
Normalized to mode
Normalized to mode
Anti-CD133
CD133
CD133
(B)
10% FBS
1% FBS
DAPI
CD133
Merged
Scale bars: 50 mm
*
### Chart
| Category | |
|---|---|
| 10% FBS | 15.22638888888889 |
| 1% FBS | 21.71625 |36
