# Supplementary material for: CD133 prevents colon cancer cell death induced by serum deprivation through activation of Akt‐mediated protein synthesis and inhibition of apoptosis
Source: FEBS Open Bio. 2021 Mar 28;11(5):1382–94. doi: 10.1002/2211-5463.13145 (PMC8091590; doi:10.1002/2211-5463.13145)
Supplement: Supplementary file 4 — Fig. S4. The activated Akt has an undetectable effect on the phosphorylation level of p38 MAPK. Immunoblot analysis. The indicated cells were cultured in 1% fetal bovine serum‐containing medium for 3 days. Cell lysates (50 µg per lane) were prepared and processed for immunoblotting with the indicated antibodies. Actin was used as a loading control. [file FEB4-11-1382-s001.pptx]

## Slide 1
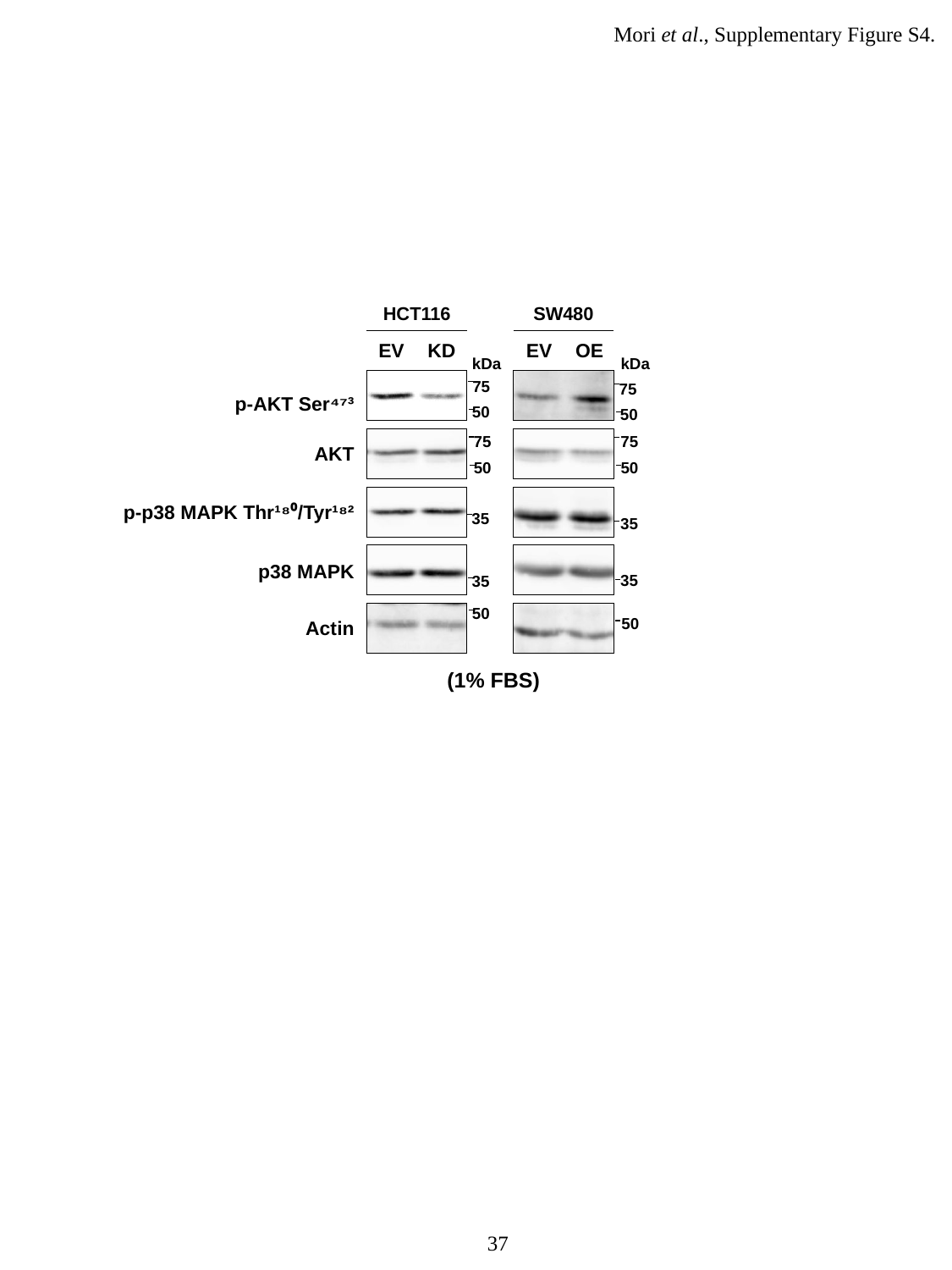

Mori et al., Supplementary Figure S4.
HCT116
SW480
EV
KD
EV
OE
kDa
kDa
75
75
p-AKT Ser⁴⁷³
50
50
75
75
AKT
50
50
p-p38 MAPK Thr¹⁸⁰/Tyr¹⁸²
35
35
p38 MAPK
35
35
50
50
Actin
(1% FBS)
37
