# Supplementary material for: CD133 prevents colon cancer cell death induced by serum deprivation through activation of Akt‐mediated protein synthesis and inhibition of apoptosis
Source: FEBS Open Bio. 2021 Mar 28;11(5):1382–94. doi: 10.1002/2211-5463.13145 (PMC8091590; doi:10.1002/2211-5463.13145)
Supplement: Supplementary file 5 — Fig. S5. Tyrosine phosphorylation of CD133 suppresses cell death in response to serum deprivation through activation of the Akt‐Bad pathway. (A) Forced expression of CD133 and its amino acid‐substituted mutants. SW480 cells were transduced with the lentiviral vector for wild‐type CD133 (SW480/OE), a phenylalanine‐substituted CD133 mutant (SW480/FF), a glutamate‐substituted CD133 mutant (SW480/EE) or with empty control vector (SW480/EV) and, finally, puromycin‐resistant cells were established. Their expression levels were checked by flow cytometry. Representative histograms are shown. (B) Trypan blue dye exclusion assay. SW480/EV (EV, open bars) SW480/OE (OE, grey bars) SW480/FF (FF, hatched bars) or SW480/EE (EE, closed bars) cells were cultured in medium containing 10% or 1% fetal bovine serum. Five days after cultivation, floating and attached cells were harvested and processed for a trypan blue assay. Data represent the mean ± SD (n = 3) and asterisks indicate a statistically significant difference (P < 0.05, ANOVA). NS, not significant. (C and D) Immunoblot analysis. The indicated cells were cultured in the presence of 1% fetal bovine serum for 3 days. Cell lysates (30 µg per lane) were prepared from floating plus attached cells (C) or from attached cells (D) and then processed for immunoblotting with the indicated antibodies. Arrows indicate cleaved PARP and caspase‐9 (C). Actin was used as a loading control. [file FEB4-11-1382-s005.pptx]

## Slide 1
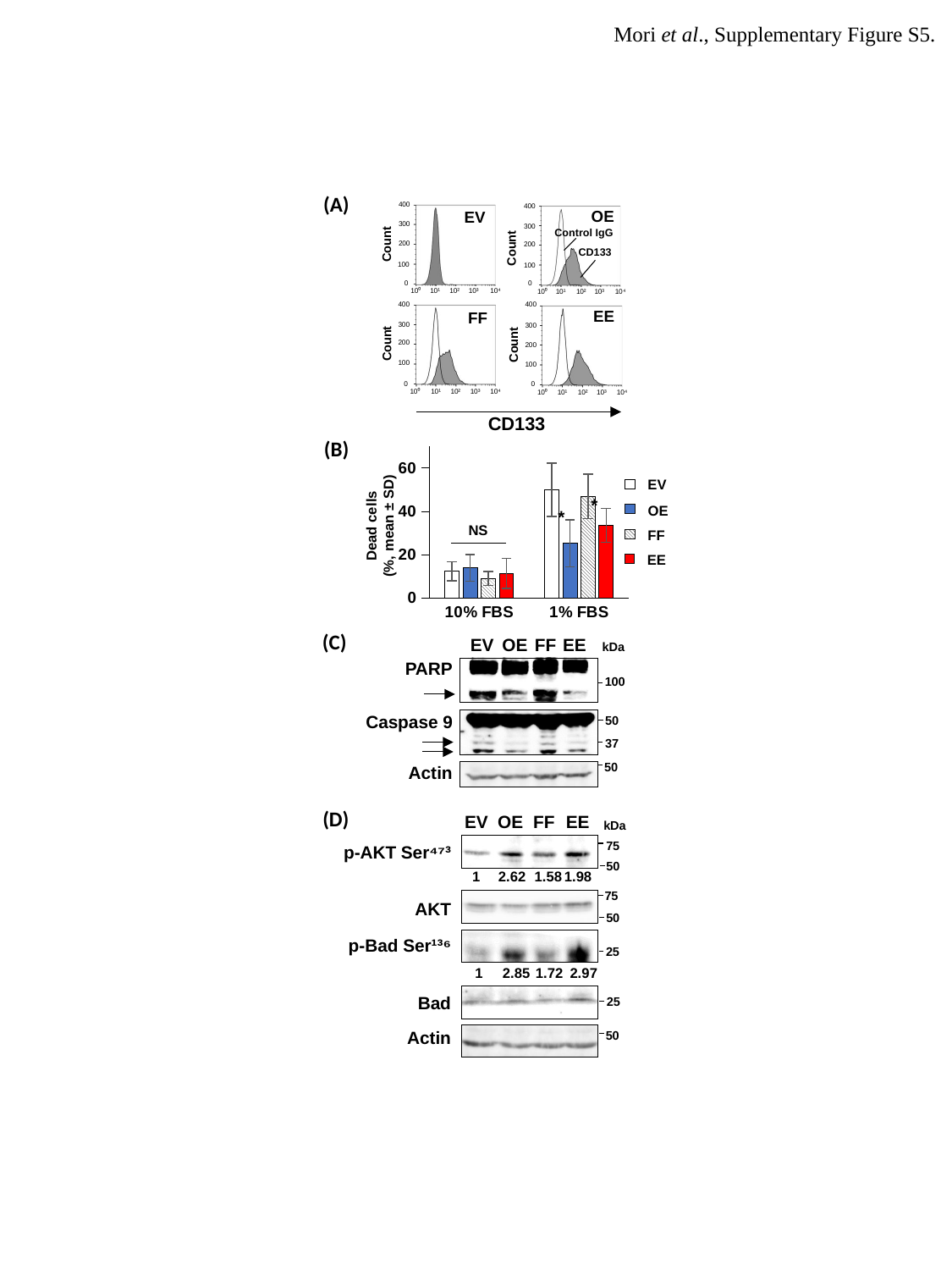

Mori et al., Supplementary Figure S5.
(A)
400
300
200
100
0
EV
Count
10⁰
10¹
10²
10³
10⁴
400
300
200
100
0
OE
Control IgG
Count
CD133
10⁰
10¹
10²
10³
10⁴
400
300
200
100
0
FF
Count
10⁰
10¹
10²
10³
10⁴
400
300
200
100
0
EE
Count
10⁰
10¹
10²
10³
10⁴
CD133
(B)
### Chart
| Category | Empty | CD133-WT | CD133-FF | CD133-EE |
|---|---|---|---|---|
| 10% FBS | 12.45118586946304 | 13.969106006053394 | 9.092318024133455 | 11.480380993202632 |
| 1% FBS | 49.908777246555346 | 25.326398586877445 | 47.00185510740627 | 33.686252006515716 |EV
OE
FF
EE
*
*
Dead cells
(%, mean ± SD)
NS
(C)
EV
OE
FF
EE
kDa
PARP
100
Caspase 9
50
37
50
Actin
(D)
EV
OE
FF
EE
p-AKT Ser⁴⁷³
1
2.62
1.58
1.98
AKT
p-Bad Ser¹³⁶
1
2.85
1.72
2.97
Bad
Actin
kDa
75
50
75
50
25
25
50
