# Supplementary figures and images for: CD133 prevents colon cancer cell death induced by serum deprivation through activation of Akt‐mediated protein synthesis and inhibition of apoptosis
Source: FEBS Open Bio. 2021 Mar 28;11(5):1382–94. doi: 10.1002/2211-5463.13145 (PMC8091590; doi:10.1002/2211-5463.13145)

## Slide 1
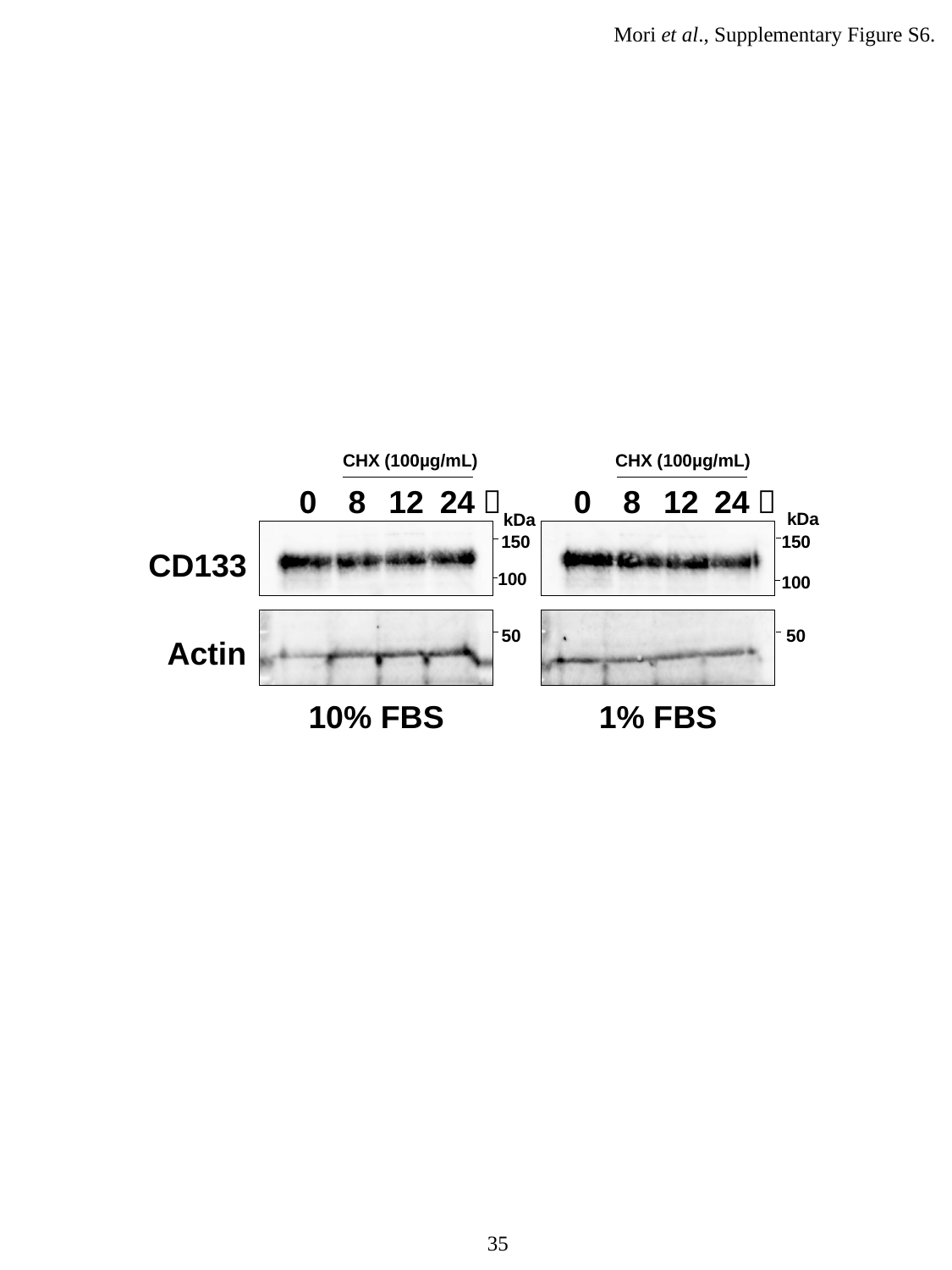

Mori et al., Supplementary Figure S6.
CHX (100µg/mL)
CHX (100µg/mL)
0
8
12
24ｈ
0
8
12
24ｈ
kDa
kDa
150
150
CD133
100
100
50
50
Actin
10% FBS
1% FBS
35

Supplement: Supplementary file 6 — Fig. S6. Serum deprivation has an undetectable effect on the half‐life of CD133. Cycloheximide block. HCT116 cells cultured in 10% fetal bovine serum‐ or 1% fetal bovine serum‐containing medium were treated with 100 µg·mL−1 cycloheximide (CHX). At the indicated time points after treatment, cell lysates (35 µg per lane) were prepared and subjected to immunoblotting with the indicated antibodies. Actin was used as a loading control. [file FEB4-11-1382-s004.pptx]
