# Supplementary material for: CD133 prevents colon cancer cell death induced by serum deprivation through activation of Akt‐mediated protein synthesis and inhibition of apoptosis
Source: FEBS Open Bio. 2021 Mar 28;11(5):1382–94. doi: 10.1002/2211-5463.13145 (PMC8091590; doi:10.1002/2211-5463.13145)
Supplement: Supplementary file 7 — Fig. S7. Hypoxia increases the CD133 level in HCT116 cells at the protein level. (A) CD133 level under hypoxia. HCT116 (6 × 10^5^ cells per dish) cells were seeded in 6‐cm culture dishes with 10% fetal bovine serum/DMEM and then cultured under hypoxic conditions (0.1% O2 and 5% CO2) using an AnaeroPack (Mitsubishi Gas Chemical, Tokyo, Japan). Twenty‐four hours after incubation, cells were lysed with a lysis buffer containing 50 mm Tris‐HCl (pH 7.5), 150 mm NaCl, 1% NP‐40, 1 mm EDTA and a protease inhibitor cocktail (Calbiochem) and the CD133 level was analyzed by immunoblot analysis with antibodies against CD133 (W6C3B1; Miltenyi Biotec) followed by horseradish peroxidase‐conjugated anti‐mouse IgG (#7074; Cell Signaling Technology) and visualized as described in the Materials and methods. (B) CD133 mRNA level under hypoxic conditions. HCT116 cells were exposed to hypoxic conditions for 24 h as described in (A). Total RNA was extracted from the cells using Isogen reagent (Nippon Gene). The first‐strand cDNA was synthesized from 1 µg of total RNA using ReverTra Ace reagent (Toyobo, Osaka, Japan) and then subjected to quantitative RT‐PCR with Thunderbird reagent in accordance with the manufacturer’s instructions (Toyobo). Relative expression levels of target genes were evaluated by the 2‐ΔΔCT method compared to the level of GAPDH in HCT116 cells under normoxia as a reference sample. Data show the mean ± SD of three independent experiments and asterisks indicate a statistically significant difference compared to HCT116/EV cells (*P < 0.05, t‐test). NS, not significant. The oligonucleotide primer sets used were: CD133, 5ʹ‐ATCTGCAGTGGATCGAGTTCTCT‐3ʹ (forward) and 5ʹ‐GCGGTGGCCACAGGTTT‐3ʹ (reverse); GLUT, 5ʹ‐CTTCACTGTCGTGTCGCTGT‐3ʹ (forward) and 5ʹ‐CCAGGACCCACTTCAAAGAA‐3ʹ (Reverse); GAPDH, 5ʹ‐ATGGAAATCCCATCACCATCTT‐3ʹ (forward) and 5ʹ‐CGCCCCACTTGATTTTGG‐3ʹ (reverse). [file FEB4-11-1382-s002.pptx]

## Slide 1
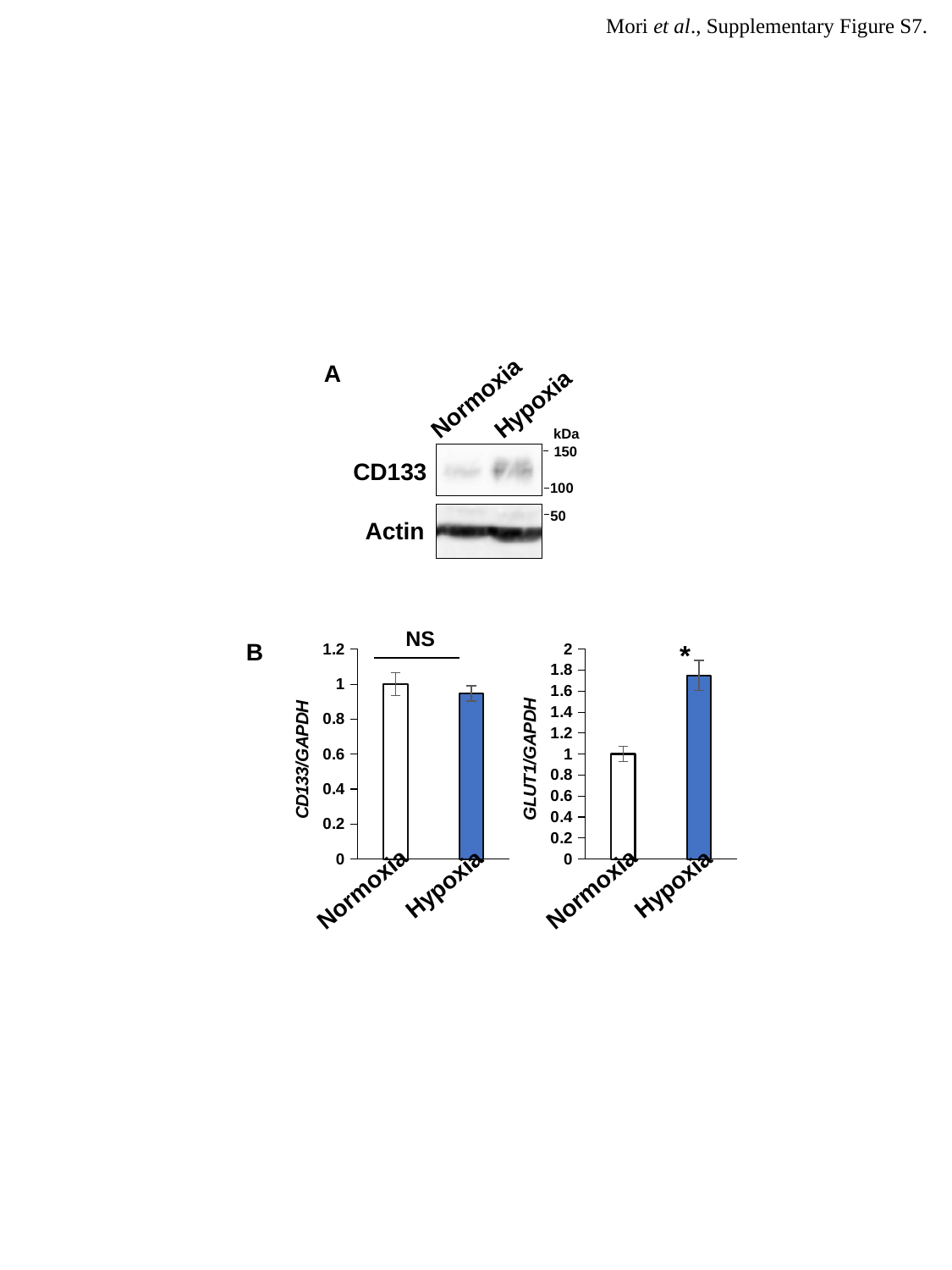

Mori et al., Supplementary Figure S7.
A
Normoxia
Hypoxia
kDa
150
CD133
100
50
Actin
NS
B
*
### Chart
| Category | |
|---|---|
| Normoxia | 1.0017768140190109 |
| Hypoxia | 1.750204468504631 |
### Chart
| Category | |
|---|---|
| Normoxia | 1.0014435972567635 |
| Hypoxia | 0.9477041904152966 |Hypoxia
Hypoxia
Normoxia
Normoxia
